# Epidemiology of chronic obstructive pulmonary disease in the global HIV-infected population: a systematic review and meta-analysis protocol

**DOI:** 10.1186/s13643-017-0467-x

**Published:** 2017-03-29

**Authors:** Jean Joel R. Bigna, Angeladine Malaha Kenne, Serra Lem Asangbeh

**Affiliations:** 1 Department of Epidemiology and Public Health, Centre Pasteur of Cameroon, PO Box 1274, Yaoundé, Cameroon; 2 Faculty of Medicine, University of Paris Sud XI, Le Kremlin Bicêtre, France; 3 Agence Nationale de Recherche sur le SIDA et les Hépatites Virales, Yaoundé, Cameroon

**Keywords:** Chronic obstructive pulmonary disease, HIV, AIDS, COPD, Epidemiology, Prevalence, Incidence, Risk factors

## Abstract

**Background:**

Evidence suggests a relationship between human immunodeficiency virus (HIV) infection and chronic obstructive pulmonary disease (COPD). Although the high burden of COPD and the HIV disease is clearly demonstrated, to the best of our knowledge, there is a lack of summary and meta-analysis data on the epidemiology of COPD in the global HIV-infected population to date. The present protocol for a systematic review and meta-analysis intends to summarize existing data on the prevalence, incidence, and risk factors of COPD in the global HIV-infected population.

**Methods and design:**

The present review will include cohort, cross-sectional, and case-control studies conducted among HIV infected people, which report prevalence, incidence, and factors associated with COPD or enough data for their estimation. We will consider published and unpublished studies in English and French language, regardless of geographical location. Relevant records will be searched using PubMed/Medline, and Scopus from inception to December 31st, 2016. Reference lists of eligible papers and relevant review articles will be screened. Two investigators will independently screen, select studies, and extract data, with discrepancies resolved by consensus or arbitrarily by a third investigator. Risk of bias and methodological quality of the included studies will be assessed using the Newcastle-Ottawa Scale. Funnel-plots and Egger’s test will be used to determine publication bias. The study-specific estimates will be pooled through a random-effects meta-analysis model to obtain an overall summary estimate. To keep the effect of studies with extremely small or extremely large estimates on the overall estimate to a minimum, the variance of the study-specific prevalence/incidence will be stabilized with the Freeman-Tukey single arc-sine transformation. The heterogeneity will be evaluated by the χ^2^ test on Cochrane’s Q statistic. Results will be presented by geographic region and by antiretroviral therapy status. We plan to summarize data on factors associated with COPD in narrative format.

**Discussion:**

This systematic review and meta-analysis will give an overview of the epidemiology of COPD in the global HIV population to inform policy-makers and to provide accurate data that can underpin effective interventions for optimizing their detection and management.

**Systematic review registration:**

PROSPERO CRD42016052639.

**Electronic supplementary material:**

The online version of this article (doi:10.1186/s13643-017-0467-x) contains supplementary material, which is available to authorized users.

## Background

Chronic obstructive pulmonary disease (COPD) is a major public health concern. This non-communicable chronic disease is projected to be the fifth in terms of disease burden by 2030 [1]. This preventable and non-curable disease is usually associated with chronic inflammatory responses in the airways, and the lung noxious particles or gases. It is characterized by persistent and progressive airflow limitation [1]. There are two mechanisms involved in the occurrence of COPD: small pulmonary airways disease (obstructive bronchiolitis) and parenchymal destruction (emphysema). The degree of mixture of these two mechanisms depends on each individual [1].

Globally, the total number of estimated COPD related deaths was about 3 million in 2015 (5% of total deaths globally) [2]. The primary cause of COPD is tobacco consumption (including second hand smokers and passive exposure) [1, 2], but evidence suggests a relationship to human immunodeficiency virus (HIV) infection [3–8]. Globally, in 2030, HIV disease is projected to be the third cause of death followed by COPD that takes the fourth place [9]. At the end of 2015, there were 37 million people living with HIV worldwide among which 1.1 million of HIV-related deaths being notified [10]. Although the high burden of COPD and the HIV disease is clearly demonstrated, to the best of our knowledge, there is a lack of summary and meta-analysis data on the epidemiology of COPD in the global HIV-infected population to date.

We hereby present the protocol for a systematic review and meta-analysis to summarize existing data on the prevalence, incidence, and risk factors of COPD in the global HIV-infected population.

### Review questions

1. What is the prevalence of COPD in the global HIV-infected population?
2. What is the incidence of COPD in the global HIV-infected population?
3. Is the prevalence or incidence of COPD higher in the global HIV-infected population compared to general population?
4. What are the factors associated with COPD in the global HIV-infected population?

### Objectives

To respond to the review questions, a systematic-review and meta-analysis will be conducted. The objectives will be:

1. To synthetize the prevalence of COPD among the global HIV-infected population;
2. To synthetize the incidence of COPD among the global HIV-infected population;
3. To compare the prevalence and incidence of COPD in the global HIV-infected population to those in the general population;
4. To synthetize data on factors associated with COPD in the global HIV-infected population.

## Methods and design

Centre for Reviews and Dissemination recommendations will be used as guidelines to conduct this review [11]. The guidelines for meta-analyses and systematic reviews of Preferred Reporting Items for Systematic Review and Meta-Analysis (PRISMA) will serve as the template for reporting the present review [12]. For the present protocol, the PRISMA for protocol was used for the reporting [13]. An additional file shows the PRISMA for protocol checklist [see Additional file 1]. This review protocol is registered in the PROSPERO International Prospective Register of systematic reviews, registration number CRD42016052639.

### Criteria for considering studies for review

#### Inclusion criteria

1. Observational studies (cross-sectional, case-control, or cohort studies) reporting the prevalence, incidence, and factors associated with COPD among HIV-infected people and/or reporting the prevalence and/or incidence of COPD in both HIV-infected and general populations; or having enough data to compute these estimates.
2. All published and unpublished studies reported from inception to December 31st, 2016 will be considered. Papers in English and French will be considered for the present review regardless of geographical limitation.

#### Exclusion criteria

1. Studies in subgroups of participants selected on the basis of the presence of COPD.
2. Case series, reviews, letters, commentaries, and editorials.
3. Studies lacking primary data and/or explicit method description.
4. Studies that are limited to other specific groups or populations like people with other respiratory chronic diseases.
5. Duplicate reports. The most comprehensive and up-to-date version will be considered for this review.
6. Studies that it will not be possible to have full text even after contacting authors.

### Search strategy for identifying relevant studies

The search strategy will be implemented in two stages:

#### Bibliographic database search

A comprehensive and exhaustive search of PubMed/Medline, and Scopus will be performed to identify all relevant articles published on COPD among HIV-infected people, and the general population from inception to December 31st, 2016, without any language restriction. A search strategy based on the combination of relevant terms will be applied. The main search strategy that will use in PubMed/Medline is shown in Table 1.

**Table 1:** PubMed search strategy for identifying studies

Search	Query
#1	(“pulmonary disease, chronic obstructive”[MeSH Terms] OR (“pulmonary”[All Fields] AND “disease”[All Fields] AND “chronic”[All Fields] AND “obstructive”[All Fields]) OR “chronic obstructive pulmonary disease”[All Fields] OR (“chronic”[All Fields] AND “obstructive”[All Fields] AND “pulmonary”[All Fields] AND “disease”[All Fields])) OR (“pulmonary disease, chronic obstructive”[MeSH Terms] OR (“pulmonary”[All Fields] AND “disease”[All Fields] AND “chronic”[All Fields] AND “obstructive”[All Fields]) OR “chronic obstructive pulmonary disease”[All Fields] OR “copd”[All Fields]) OR (“pulmonary disease, chronic obstructive”[MeSH Terms] OR (“pulmonary”[All Fields] AND “disease”[All Fields] AND “chronic”[All Fields] AND “obstructive”[All Fields]) OR “chronic obstructive pulmonary disease”[All Fields] OR (“chronic”[All Fields] AND “obstructive”[All Fields] AND “airway”[All Fields] AND “disease”[All Fields]) OR “chronic obstructive airway disease”[All Fields]) OR (“pulmonary disease, chronic obstructive”[MeSH Terms] OR (“pulmonary”[All Fields] AND “disease”[All Fields] AND “chronic”[All Fields] AND “obstructive”[All Fields]) OR “chronic obstructive pulmonary disease”[All Fields] OR “coad”[All Fields]) OR (“pulmonary disease, chronic obstructive”[MeSH Terms] OR (“pulmonary”[All Fields] AND “disease”[All Fields] AND “chronic”[All Fields] AND “obstructive”[All Fields]) OR “chronic obstructive pulmonary disease”[All Fields] OR (“chronic”[All Fields] AND “obstructive”[All Fields] AND “lung”[All Fields] AND “disease”[All Fields]) OR “chronic obstructive lung disease”[All Fields]) OR (airway[All Fields] AND flow[All Fields] AND obstruction[All Fields]) OR (chronic[All Fields] AND airway[All Fields] AND flow[All Fields] AND obstruction[All Fields]) OR (“bronchitis, chronic”[MeSH Terms] OR (“bronchitis”[All Fields] AND “chronic”[All Fields]) OR “chronic bronchitis”[All Fields] OR (“chronic”[All Fields] AND “bronchitis”[All Fields])) OR (“pulmonary emphysema”[MeSH Terms] OR (“pulmonary”[All Fields] AND “emphysema”[All Fields]) OR “pulmonary emphysema”[All Fields])
#2	(“hiv”[MeSH Terms] OR “hiv”[All Fields]) OR (“acquired immunodeficiency syndrome”[MeSH Terms] OR (“acquired”[All Fields] AND “immunodeficiency”[All Fields] AND “syndrome”[All Fields]) OR “acquired immunodeficiency syndrome”[All Fields] OR “aids”[All Fields])
#3	#1 AND #2
#4	#3 Limits: from inception to 2016/12/31

#### Searching other sources

A manual search to scan the reference lists of eligible papers and other relevant review articles will be conducted.

### Selection of studies for inclusion in the review

Two investigators will independently identify articles and sequentially screen their titles and abstracts for eligibility. Full texts of articles deemed potentially eligible will be acquired. These investigators will further independently assess the full text of each study for eligibility and consensually retain studies to be included. Disagreements when existing will be solved by a third investigator. A screening guide will be used to ensure that the selection criteria are reliably applied by all investigators. Studies selection will be managed using EndNote X7.

### Data extraction and management

Two investigators will extract data pertaining to:

1. Author details: name of first author and publication year;
2. Study characteristics: country, region, study design, setting, data source, sampling method, sample size, total person duration of follow-up, data collection period, and response rate;
3. Participants’ characteristics: age, gender, HIV related data (time since diagnosis, severity of the disease, antiretroviral (ART) regimens, and duration of treatment);
4. COPD characteristics: diagnostic criteria used, prevalence, incidence, associated factors, number of participants tested and diagnosed with COPD overall, and by subgroup of interest like level of urbanization, gender, tobacco smoking, and ART regimens.

Where only primary data (sample size and number of outcomes) will be provided, these data will be used to calculate the prevalence or incidence estimates. Data will be extracted using a preconceived, piloted, and standardized data abstraction form. Disagreements between investigators will be reconciled through discussion and consensus, or arbitration by a third investigator whenever necessary. In case of multinational studies, the results will be separated to show the estimate within individual countries. When it will not be possible to disaggregate the data by country, the study will be presented as one, and the countries in which the study was done will be shown.

### Appraisal of the quality of included studies

The Newcastle-Ottawa Scale (NOS) will be used to evaluate the methodological quality of case control and cohort studies included in this review. An additional file shows the NOS in detail [see Additional file 2] [14]. An adapted version of this NOS will be used for cross-sectional studies. These instruments will be used to also make an assessment of the risk of bias affecting study findings. There is no validation study that provides a cut-off score for rating low-quality studies; at priori, we will arbitrarily consider 0–3, 4–6, and 7–9 stars as high, moderate, and low risk of bias respectively.

### Data synthesis including assessment of heterogeneity

Data will be analyzed using Stata software (Stata Corp V.13, Texas, USA). Unadjusted prevalence/incidence and standard errors of hypertension will be recalculated based on the information of crude numerators and denominators provided by individual studies. To keep the effect of studies with extremely small or extremely large prevalence estimates on the overall estimate to a minimum, the variance of the study-specific prevalence/incidence will be stabilized with the Freeman-Tukey single arc-sine transformation before pooling the data with the random-effects meta-analysis model [15]. Heterogeneity will be evaluated by the chi-squared test on Cochrane’s Q statistic [16], which will be quantified by I-squared values, assuming that I-squared values of 25, 50, and 75% being representative of low, medium, and high heterogeneity, respectively [17]. When substantial heterogeneity will be detected, we will perform a subgroup analysis to investigate the possible sources of heterogeneity using the following grouping variables: age group, sex, study setting (rural vs urban), geographical area, tobacco consumption status (current smokers, former smokers, and never smoked), ART regimens, and study quality. Additionally, we will report estimates after adjustment on publication bias using the trim-and-fill method [18]. We will assess inter-rater agreement between investigators for study inclusion, data extraction, and methodological quality assessment using Kappa Cohen’s coefficient [19]. We presume that the reporting of factors associated with COPD will present high heterogeneity. If it is the case, we will summarize the findings in a narrative format.

### Assessment of reporting biases

Symmetry of funnel plots and Egger’s test will be done to assess the presence of publication and selective reporting bias [20]. A *p* value < 0.10 will be considered indicative of statistically significant publication bias.

### Potential amendments

We do not intend to make any amendments to the protocol, to avoid the possibility of outcome reporting bias. Any amendments during review process will be reported transparently.

## Discussion

The overarching goal of this review is to inform policy-makers on the magnitude of COPD in HIV infection and to provide accurate data that can underpin effective interventions for optimizing their detection and management. In this context, we plan to conduct this review with the aim of estimating the burden of this condition in this population, there is a need of summarized and global data on the topic. We hope that this review will serve to draw attention and raise awareness on this growing concern. To the best of our knowledge, this will be the first systematic review and meta-analysis which aims to estimate the global prevalence, incidence, and risk factors of COPD in HIV-infected people. This review would be limited by the impossibility to disaggregate data between patients on ART and those naïve of ART. Another possible limitation could be the predominance of hospital-based studies limiting the generalizability of findings.

The current study is based on data already corrected and as such ethics is not a requirement. The final report of the systematic review in the form of scientific paper will be published in peer-reviewed journals. Findings will further be presented at conferences and be submitted to relevant health authorities. We also plan to update the review in future to monitor changes and to guide health service and policy solutions.

## Additional files

Additional file 1: Preferred Reporting Items for Systematic Review and Meta-Analysis for protocol checklist. (DOCX 56 kb)

Additional file 2: Newcastle-Ottawa quality assessment scale. (PDF 135 kb)

## Abbreviations

ART: Antiretroviral treatment
COPD: Chronic obstructive pulmonary disease
FEV1: Force expiratory volume in 1 s
FVC: Forced vital capacity
HIV: Human immunodeficiency virus
NOS: Newcastle-Ottawa Scale
PRISMA: Preferred Reporting Items for Systematic Review and Meta-Analysis
